# Hydroalcoholic Leaf Extract of *Punica granatum*, alone and in Combination with Calcium Hydroxide, Is Effective against Mono- and Polymicrobial Biofilms of *Enterococcus faecalis* and *Candida albicans*

**DOI:** 10.3390/antibiotics11050584

**Published:** 2022-04-27

**Authors:** Monica Naufel Sousa, Alessandra Teixeira Macedo, Gabriella Freitas Ferreira, Haryne Lizandrey Azevedo Furtado, Aruanã Joaquim Matheus Costa Rodrigues Pinheiro, Lídio Gonçalves Lima-Neto, Valéria Costa Fontes, Rayana Larissa Pinheiro Soares Ferreira, Cristina Andrade Monteiro, Angela Falcai, Lillian Nunes Gomes, Queila da Silva Rosa Bragança, Dennyse de Sousa Brandão Torres, Lívia Câmara de Carvalho Galvão, Rodrigo Assuncao Holanda, Julliana Ribeiro Alves Santos

**Affiliations:** 1 Laboratório de Micologia, Universidade CEUMA (UNICEUMA), São Luís 65067-120, MA, Brazil; monicanaufelsousa@hotmail.com (M.N.S.); alessandra.macedo@hotmail.com (A.T.M.); haryne.lizandrey@gmail.com (H.L.A.F.); rayana-larissa@hotmail.com (R.L.P.S.F.); angela.falcai@ceuma.br (A.F.); dennyse.torres@ceuma.br (D.d.S.B.T.); raholanda@yahoo.com.br (R.A.H.); 2 Departamento de Farmácia, Campus Avançado Governador Valadares, Universidade Federal de Juiz de Fora, Governador Valadares 35010-180, MG, Brazil; gabriella.farmacia@yahoo.com.br (G.F.F.); queilabraganca@outlook.com (Q.d.S.R.B.); 3 Laboratório das Infecções do Trato Respiratório, Universidade CEUMA (UNICEUMA), São Luís 65067-120, MA, Brazil; aruanajm@hotmail.com (A.J.M.C.R.P.); lidio.neto@ceuma.br (L.G.L.-N.); valeria.cfontes@gmail.com (V.C.F.); 4 Instituto Federal do Maranhão (IFMA), São Luís 65030-005, MA, Brazil; crisand2003@yahoo.com.br; 5 Laboratório de Patologia e Biologia Molecular, Universidade Federal do Maranhão (UFMA), São Luís 65080-805, MA, Brazil; lilian.gomes20@gmail.com; 6 Laboratório de Pesquisa em Odontologia, Universidade CEUMA (UNICEUMA), São Luís 65067-120, MA, Brazil; livia.camara@ceuma.br

**Keywords:** *Candida albicans*, *Enterococcus faecalis*, *Punica granatum*, endodontic infections, antibiofilm

## Abstract

Failures in endodontic treatments are mostly associated with the difficulty in eradicating microbes of the root canal system, highlighting the need to develop novel effective antimicrobials. *Punica granatum* (pomegranate) leaf hydroalcoholic extract may be a potential alternative in canal dressing, owing to its antimicrobial properties. The objective of this study was to evaluate the antimicrobial activity of hydroalcoholic leaf extract of *Punica granatum* (HEPg) alone or in combination with calcium hydroxide (Ca(OH)_2_) against *Enterococcus faecalis* and *Candida albicans* in isolation and in mono- and polymicrobial biofilms. Microdilution tests in broth and assays for inhibition of biofilm formation were carried out to evaluate the antimicrobial properties of HEPg and HEPg + Ca(OH)_2_ against *Enterococcus faecalis* and *Candida albicans*. The cytotoxicity of HEPg in HaCaT cells was evaluated by MTT assay. HEPg and HEPg + Ca(OH)_2_ exerted significant antimicrobial activity against planktonic cells and mono- and polymicrobial biofilms. The combination of *Punica granatum* extract with Ca(OH)_2_ appears to be a promising alternative in endodontic treatments, which could be tested in vivo to confirm the efficacy of this mixture in disinfecting root canal systems.

## 1. Introduction

Failures in root canal treatments are usually attributed to the inefficacy of completely eliminating the canal microbiota [1,2,3] persistent in endodontic infections [4,5]. In this context, *Enterococcus faecalis* and *Candida albicans* deserve special attention, as they have been repeatedly isolated from intra-radicular persistent infections [1,2,6]. *E. faecalis* and *C. albicans* are opportunistic pathogens found in polymicrobial infections, the gastrointestinal tract, oral cavity, and other non-sterile sites such as soil, sewage, water, and food [7,8,9]. They use dentin as a source of nutrition and interact with other microorganisms to form a complex biofilm that makes them resistant to antimicrobial agents [10]. 

*E. faecalis* is a Gram-positive coccus and facultative anaerobe. Several virulence and resistance factors contribute to its survival in the root canal. When this microorganism infects the tooth, the connective tissue fluids underlying the alveolar bone and the periodontal ligament are used as a substrate for colonizing the root canal system. Another virulence factor is its ability to produce lytic enzymes, cytolysins, aggregation substances, and lipoteichoic acid [1,11,12]. *E. faecalis* forms biofilms in medicated root canals and has intrinsic (against calcium hydroxide and sodium hypochlorite) and acquired (against macrolides) [13] antimicrobial resistance.

*C. albicans* is commonly detected in primary and refractory endodontic infections. This yeast produces proteolytic enzymes to switch its form to blastoconidia, germ tubes, true hyphae, pseudohyphae, and chlamydoconidia, which contribute to its colonization and persistence. These virulence factors are linked with the failure to sanitize the root canal [7]. 

Endodontic therapy has different stages to promote the disinfection of the root canal. The canal dressing phase aims to eliminate microorganisms that were not removed in the biomechanical preparation phase. Calcium hydroxide (Ca(OH)_2_) is widely used in delay dressing to treat the canal systems [6]. It was originally introduced to the field of endodontics as a pulp-capping agent, since its main actions result from the ionic dissociation of the Ca^2+^ and OH^−^ ions inducing hard tissue deposition and antimicrobial effects. The release of hydroxyl ions (OH^−^) into the aqueous medium has the potential to damage the bacterial cytoplasmic membrane, denature the proteins, and damage the DNA [4]. However, *E. faecalis* uses a proton pump mechanism to resist the action of OH^−^ ions and maintains an acidic pH inside the bacterial cell, thereby preventing the antimicrobial action of Ca(OH)_2_, a strong base [2].

Several products have been used to avoid failures in root canal treatment. However, natural products have contributed significantly to the discovery of chemical structures to create new medicaments to be used as innovative therapeutic agents against this prevalent disease [14]. Natural products have been gaining ground in medical therapy, as they often present: (1) low risk and toxicity; (2) high efficacy and reproducibility; (3) consistent quality; and (4) the possibility of potentiating allopathic medicines [15]. For this reason, it is important that extensive studies involving sources of natural medicines are carried out. In addition, the combination of compounds usually requires lower doses of both. This reduction might lead to a decrease in toxicity and an augmentation of the efficacy and speed of action, enhancing the antimicrobial activity [16].

*Punica granatum* (Pg) is known for its anti-inflammatory properties as seen in induced models of peritonitis [17] and acute lung injury [18]. Recently, we have observed the potential antifungal activity against biofilms of yeast such as *Cryptococcus gattii* and *C. laurentii* [19], while Álvarez-Martínez et al. [20] observed antibacterial activity. These studies encouraged us to use Pg extract as an effective alternative for endodontic treatment against oral pathogens. Therefore, the aim of this present study was to evaluate the antimicrobial activity of hydroalcoholic leaf extract of *Punica granatum* (HEPg) alone or in association with calcium hydroxide against *E. faecalis* and *C. albicans* in isolation and in mono- and polymicrobial biofilms.

## 2. Results

### 2.1. Phytochemical Analysis of Punica Granatum Leaf (PgL)

Phytochemical analyses of the hydroalcoholic leaf extract of *Punica granatum* (HEPg) were evaluated by the analysis of color intensity and/or precipitate formation, and revealed the presence of coumarins, flavonoids (such as xanthones, flavone, flavonol, and flavanone), and phenolic acids as described previously by Marques et al. [17].

### 2.2. Characterization of the Antimicrobial Effects of the Hydroalcoholic Leaf Extract of Punica granatum (HEPg)

Evaluation of the antimicrobial effects of HEPg showed a minimum inhibitory concentration (MIC) of 62.50 µg/mL for *C. albicans* and 15.62 µg/mL for *E. faecalis*. Ca(OH)_2_ MIC values were 125 µg/mL for both the microorganisms. The MIC for NYST was 16 µg/mL and 0.01 µg/mL for AMX. Minimum fungicidal concentration (MFC) was 4000 µg/mL for *C. albicans* and minimum bactericidal concentration (MBC) was 2000 µg/mL for *E. faecalis* for both the conditions.

### 2.3. Hydroalcoholic Leaf Extract of Punica granatum (HEPg) Demonstrates Biofilm Elimination Activity

**Figure 1:**
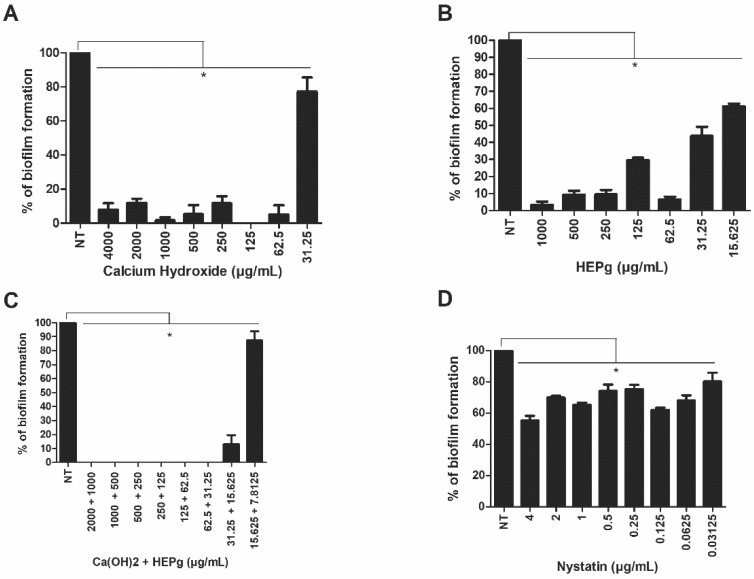
Activity of calcium hydroxide (Ca(OH)_2_) (**A**); hydroalcoholic leaf extract of *Punica granatum* (HEPg) (**B**); calcium hydroxide (Ca(OH)_2_) + hydroalcoholic leaf extract of *Punica granatum* (HEPg) (**C**); and Nystatin (**D**) against *Candida* biofilm. * = *p* < 0.05 at different concentrations that inhibit biofilm formation of *Candida albicans* compared to cells not treated (NT). Data are given as mean ± standard deviation (SD).

**Figure 2:**
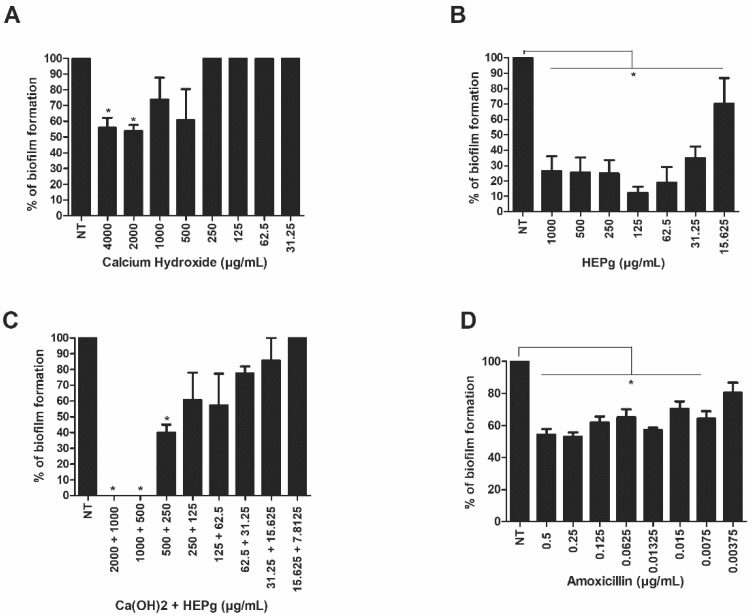
Activity of calcium hydroxide (Ca(OH)_2_) (**A**); hydroalcoholic leaf extract of *Punica granatum* (HEPg) (**B**); calcium hydroxide (Ca(OH)_2_) + hydroalcoholic leaf extract of *Punica granatum* (HEPg) (**C**); and Amoxicillin (**D**) against *Enterococcus* biofilm. * = *p* < 0.05 at different concentrations that inhibit biofilm formation of *E. faecalis* compared to cells not treated (NT). Data are given as mean ± standard deviation (SD).

**Figure 3:**
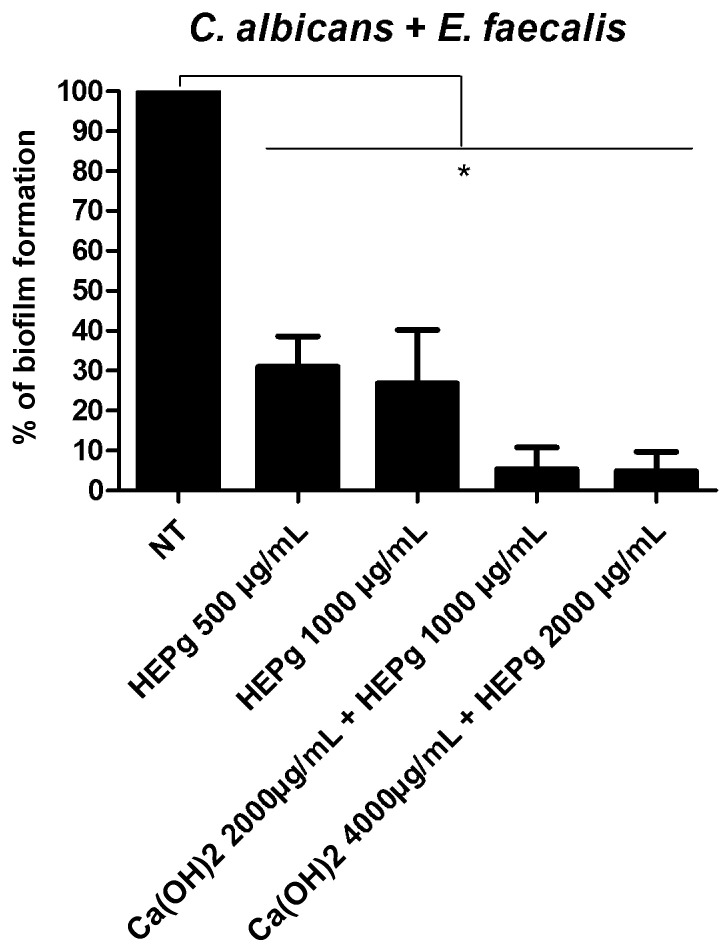
Activity of hydroalcoholic leaf extract of *Punica granatum* (HEPg); calcium hydroxide (Ca(OH)_2_) + hydroalcoholic leaf extract of *Punica granatum* (HEPg) against polymicrobial biofilm formation. * = *p* < 0.05 at different concentrations that inhibit biofilm formation of *C. albicans* plus *E. faecalis* compared to cells not treated (NT). Data are given as mean ± standard deviation (SD).

Our results demonstrate that Ca(OH)_2_ at 62.50 µg/mL was able to impair the formation of *C. albicans* biofilms (Figure 1A), but not *E. faecalis* biofilms (Figure 2A). Interestingly, HEPg at 15.62 µg/mL was able to significantly inhibit monomicrobial biofilms composed of *C. albicans* (Figure 1B) or *E. faecalis* (Figure 2B). The combination of Ca(OH)_2_ and HEPg inhibited *C. albicans* biofilm formation at all the concentrations tested (Figure 1C) (*p* < 0.05) and *Enterococcus* biofilm formation at 500 µg/mL + 250 µg/mL (Figure 2C). Nystatin impaired yeast biofilm formation at 0.03125 µg/mL (Figure 1D) and amoxicillin impaired bacterial biofilm formation at a concentration of 0.0075 µg/mL (Figure 2D). Polymicrobial biofilm formation was inhibited by HEPg alone (500 and 1000 µg/mL), Ca(OH)_2_ (2000 µg/mL) plus HEPg (1000 µg/mL) and Ca(OH)_2_ (4000 µg/mL) plus HEPg (2000 µg/mL) (Figure 3).

### 2.4. Cytotoxicity Assays

**Figure 4:**
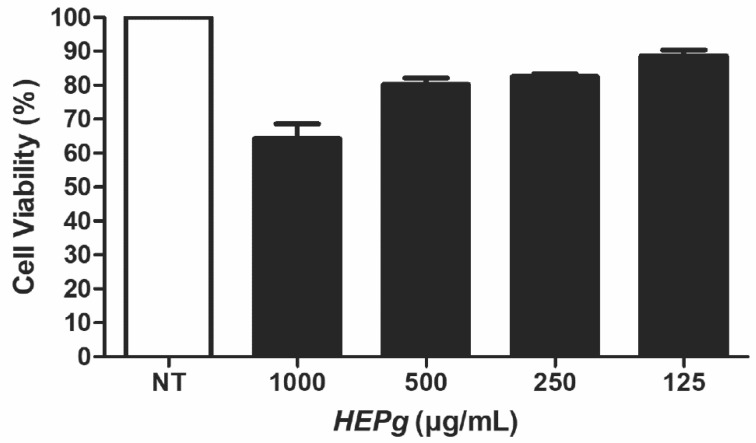
Cytotoxicity of hydroalcoholic leaf extract of *Punica granatum* (HEPg) against HaCaT cells at concentrations tested. HaCaT cells were treated with 1000, 500, 250, and 125 µg/mL of the hydroalcoholic leaf extract of *Punica granatum* (HEPg). The results were expressed as percentage of cellular viability. NT = no treatment. Data are given as mean ± standard deviation (SD).

The cell viability was more than 80% for concentrations of HEPg that ranged from 125 to 500 µg/mL, as revealed by the MTT assay. At 1000 µg/mL HEPg, approximately 70% of cells were viable (Figure 4).

## 3. Discussion

Studies evaluating the therapeutic effects of the leaves of *Punica granatum* are very few and limited to demonstrating its antioxidant, anti-inflammatory, anti-cholinesterase, and cytotoxic activities [17,18,21]. Antimicrobial properties have been majorly associated with other parts of the plant, such as the flower, fruit, root or peel [22,23,24]. Owing to the abundance of the leaf compared to the flower or fruit, it is interesting to study its biological properties.

Previous studies demonstrated that HEPg contains several classes of bioactive compounds. The phytochemical screening of hydroalcoholic extract from *Punica granatum* leaves revealed the presence of coumarins, flavonoids (such as xanthones, flavone, flavonol, and flavanone) and phenolic acids. Other secondary metabolite classes examined were not detected in the HEPg [17,18].

*C. albicans* and *E. faecalis* are members of the normal human gut microbiota [25]. These species are typical etiologic agents of endodontic infections [7,11]. The common presence of *E. faecalis* in root canals of those with treatment failures suggests that this microbe is an obstacle to the successful outcome of endodontic treatment [26].

The inhibitory concentrations of *Punica granatum* leaf extract against *C. albicans* and *E. faecalis* were below 100 µg/mL, indicating its potential antimicrobial properties [27], since natural products are considered strong inhibitors of microbial activity, when MIC values are lower than 500 μg/mL [14,28]. These data confirm the findings of previous studies carried out using other parts of the plant against *Candida* spp. Researchers described a pericarp extract that presented an MIC of 125 mg/mL to *C. parapsilosis* and *C. albicans*, and 62.5 μg/mL to *C. utilis*, *C. lusitaniae* and *C. glabrata* [29]. However, hydroalcoholic extract of flowers of *P. granatum* displayed a very high MIC that ranged from 50,000 µg/mL to 125,000 µg/mL against *E. faecalis* [23,30]. An important factor that affects the MIC is the difference in the composition of extracts, which in turn is influenced by the plant part it is collected from, season of collection, age of the plant, development, organization, strategy of drying, and extraction procedure [31]. Álvarez-Martínez et al. [20] showed that the *P. granatum* extract mainly contained hydrolysable tannins such as punicalin and punicalagin as the main antimicrobial compounds.

Marques et al. [17] showed that the chemical composition of the acetate fraction, obtained from HEPg, contains 3,3′-di-O-methylellagic acid, kaempferol, and kaempferol 3-O-glycoside. In addition, our group also showed antimicrobial activity of the acetate fraction of *P. granatum* leaf extract against environmental and clinical isolates of *Cryptococcus* [19].

Researchers observed that polymicrobial biofilms formed by yeasts such as *C. albicans* and Gram-positive and/or Gram-negative bacteria are associated with an increased tolerance to different antibiotics [32].

Owing to its inhibitory effect on *C. albicans* and *E. faecalis*, leaf extract of *Punica granatum* shows a promising use in controlling biofilms formed on the root canal. The results observed with the *Punica granatum* leaf extracts against *C. albicans* biofilms are possibly due to some active principle in the leaves which stimulated the investigation of its antimicrobial activity, similar to that seen with the fruit peel against this yeast, reported morphological changes, irregularities in the hyphae and membrane, where the cell wall became thicker and densely electric, and still influenced cell aggregation and inhibition of yeast growth [29]. Interestingly, *P. granatum* L. flower water extract had a significant effect on reducing *E. faecalis* biofilm formation on orthodontic wire. The authors proposed that *P. granatum* L. flower water extract can prevent primary colonization and adhesion of microorganisms [30].

An important stage in endodontic therapy is the root canal dressing. Calcium hydroxide (Ca(OH)_2_), mostly used as dressing in treatment of dental canals, has low solubility, diffusion, and antimicrobial activity [6]. However, the eradication of bacteria within the root canals is not always successful [11]. The MIC of Ca(OH)_2_ when used along with the extract of *Punica granatum* was lower than those of the individual compounds when tested against *C. albicans* and *E. faecalis*, as well as against mono/polymicrobial biofilms. The mixture is effective at low concentration of Ca(OH)_2_.

It is important to note that polymicrobial interaction studies involving *C. albicans* and *E. faecalis* revealed that the presence of *E. faecalis* inhibited the formation of *C. albicans* hyphae in an alternative model, concluding that this particular association is harmful to *Candida* [8]. However, an opposite point of view advocates that the increase in virulence in relation to polymicrobial biofilms, composed of Gram-positive and Gram-negative bacteria and *C. albicans*, appears to be related to a peculiarity of the first work, which had only considered the interaction between *C. albicans* and *E. faecalis* [32].

The extracts of *Punica granatum* displayed low cytotoxicity at the concentrations that were effective against the microbes. Further, the association between *Punica granatum* extract and Ca(OH)_2_ appears to be a potential alternative to endodontic treatments that may be tested in vivo to clarify the efficiency of this mixture for sanitization of root canal systems.

We suggest that further research investigating its mechanism of action and in vivo analysis is essential to better define the antimicrobial activity of *Punica granatum* leaf and clinical application of this natural product.

## 4. Materials and Methods

### 4.1. Preparation of Hydroalcoholic Extract from the Leaves of Punica granatum L.

Leaves of *Punica granatum* L. were collected at the Ático Seabra Herbarium of the Universidade Federal do Maranhão in São Luís, Maranhão, Brazil and a voucher specimen was deposited (voucher number 01002). The leaves were air-dried at 40 °C for 18 days and then ground in powder. The powder (186.4 g) was mixed with 70% ethanol for 7 days with occasional agitation at room temperature. The hydroalcoholic leaf extract of *Punica granatum* L. (HEPg) was obtained by concentration through rotary evaporation and drying and then the lyophilized material was stored at −80 °C until use [17]. The extract of *Punica granatum* L. was previously characterized in the studies of Marques et al. [17] and Pinheiro et al. [18] and was kindly provided by Prof Dr. Lídio Gonçalves Lima Neto (UNICEUMA). For the antifungal test, the HEPg powder was diluted in distilled water to a concentration of 1000 µg/mL.

### 4.2. Strains

*Enterococcus faecalis* ATCC 19433 and *Candida albicans* ATCC 90028 were used for antimicrobial and biofilm formation assays and were grown on Mueller–Hinton agar and Sabouraud agar plates at 37 °C for 48 h, respectively, before the initiation of experiments.

### 4.3. Inoculum Preparation

Inocula of 5.0 × 10^5^ CFU/mL (*E. faecalis*; Mueller–Hinton broth Difco, Detroit, MI, USA) and 1.0 × 10^3^ CFU/mL of *C. albicans* (in RPMI-1640 buffered with MOPS Sigma–Aldrich, St Louis, MO, USA) were prepared.

### 4.4. Determination of Minimum Inhibitory Concentration (MIC)

Minimum inhibitory concentrations were determined using the broth microdilution method, as described in Clinical and Laboratory Standards Institute M27-A3 and M07-A10 [33,34]. The drug concentrations ranged from 15.62 to 4000 µg/mL for HEPg and Ca(OH)_2_. AMX (0.0075 to 4 µg/mL) was also tested for *E. faecalis* and NYST (0.015 to 16 µg/mL) for *C. albicans*. The plates were incubated under aerobic conditions at 37 °C for 24 h. The MIC was defined as the lowest concentration that completely inhibited microbial growth, which was indirectly assessed by enzymatic reduction of 3-(4,5-dimethylthiazol-2-yl)-2,5-diphenyl-2H-tetrazolium bromide (MTT) to formazan salt. Briefly, a concentration of 0.5 mg/mL of MTT (Sigma–Aldrich, St Louis, MO, USA) was adjusted per well and the plates were incubated at 37 °C for 3 h. Formazan salt was solubilized in DMSO before spectrophotometric reading at 570 nm. Controls of growth (microbial culture without addition of drugs) and medium (culture media without microbial addition) were used as a reference for the live and dead cells, respectively, based on enzymatic reduction.

### 4.5. Determination of Minimal Bactericidal and Fungicidal Concentrations

Minimal Bactericidal Concentration (MBC) and Minimal Fungicidal Concentration (MFC) assays were conducted following the CLSI method for each strain. Aliquots of 3 μL from each well with a negative result (indicating no growth) were plated and incubated for 48 h at 37 °C. The MBC and MFC were considered as the lowest concentration that totally prevented growth.

### 4.6. Inhibition Test of Preformed Mono- or Poly-microbial Biofilms Composed of Enterococcus faecalis and Candida albicans

Quantitative biofilm measurements were conducted as described previously [35]. For mono-microbial biofilms, MIC concentrations were used, while for poly-microbial biofilms, the following concentrations were used: 500 µg/mL of HEPg; 1000 µg/mL of HEPg; 2000 µg/mL HEPg and 4000 µg/mL Ca(OH)_2_; and 1000 µg/mL HEPg and 2000 µg/mL Ca(OH)_2_. The plates were incubated under aerobic conditions at 37°C for 24 h, washed three times with 200 μL/well with sterile phosphate-buffered saline (PBS 1×; pH 7.2) and fixed with 200 μL/well of methanol for 15 min at room temperature. Two washes with 1XPBS were carried out before the addition of 200 μL of crystal violet stain. The plates were incubated at 37 °C for 15 min, washed, dried, fixed with 250 μL of ethanol and read using a spectrophotometer at 540 nm. The results were tabulated and the means of the growth percentage (ratio between test/control x 100) were calculated for the statistical analysis.

### 4.7. Cytotoxicity Effects of HEPg in HaCaT cells

HaCaT cells (3 × 10^5^ cells/mL) were grown in 96-well flat-bottom plates containing RPMI-1640 (Sigma–Aldrich, St Louis, MO, USA) with 10% fetal bovine serum and 1% antibiotics (10,000 μg/mL streptomycin and 10,000 units/mL penicillin) supplemented with different concentrations of HEPg (ranging from 1000 to 125 µg/mL). The plates were incubated at 37 °C with 5% CO_2_. The MTT assay was performed to evaluate the cell viability as previously described [36]. The results were expressed as a percentage of cellular cytotoxicity, and this was calculated from the following formula:

**%** Cytotoxicity = [1 − (Test Absorbance/Blank Absorbance)] * 100 (1)

All the data were compared with the negative control group, composed only of RPMI complete medium and HaCaT cells.

### 4.8. Statistical Analysis

Results are presented as mean ± standard deviation. Statistical analyses of the data were performed using GraphPad Prisma version 5.0. The results were evaluated by the Analysis of Variance (ANOVA) test and Friedman’s non-parametric tests and Student’s *t*-test. The value of *p* <0.05 was considered significant.

## Data Availability

Data are contained within the article.
